# Propriety of Bleeding in Acute Disease

**Published:** 1876-06

**Authors:** 


### PROPRIETY OF BLEEDING IN ACUTE DISEASE.

Mr. J. T. Mitchell, who has for more than thirty years filled the office of medical director in a very large insurance company, and whose duty it has been to record the cases of death and their causes, has been struck with the frequent instances in which death has occurred from acute pleuro-pneumonia, peritonitis, and other inflammatory attacks of vital organs, in subjects, many of whom were young, and before their illness enjoyed robust health, and has been led to the conclusion that this great mortality has arisen from the neglect of general and free bleeding in the early stages of such affections. He willingly admits there was a time when bleeding was carried to an injurious extent, and that it is only at a very early period that this remedy can be so advantageously employed, but he declares his conviction that nothing which he has observed in the extensive field of public and private practice, now protracted as student and practitioner beyond sixty years, has ever shown him that the abstraction of blood under the circumstances described has ever done harm, or has not been the most ready and efficient means of cure.

He relates the following striking example, which occurred during an epidemic of influenza in Lambeth, where he was at the time a district medical officer:

"'One morning, whilst he (Mr. M.'s assistant) and I were engaged among a crowd of waiting people, a young woman, in a most excited state, rushed unceremoniously into the surgery, pushing the people aside, and with great importunity came up to me exclaiming, 'Oh, sir, do come as soon as possible to see my husband, for he is dying!' I asked her from what he was suffering, his age, and his business, when she answered, 'He is a carter, and about twenty-six years of age; he was quite well the day before yesterday until night, when he was seized with difficulty in breathing, a dreadful cough, and agonizing pain in the side; his face is now perfectly blue, and his hands and feet are as cold as ice.' As her importunity was so great, I said to my assistant, 'This poor fellow evidently is suffering from acute pleuro-pneumonia; go down and immediately take from his arm twelve or sixteen ounces of blood.' Upon which he said, 'I never bled in my life, and I have not a lancet.' I then gave him a lancet and a short lecture on bleeding, and sent him off with the poor woman: very soon after which he returned, and told me that 'the poor man was dying, and nothing would save him—indeed, he was pulseless and cold.' As soon as we had dismissed the cases surrounding us, we proceeded together to this patient's house, where I found him suffering in the manner described by his wife and the assistant. I had seen cases much in the same state, but perhaps never under the same extremely alarming circumstances. His wife now repeated what she had told me before of his previous condition, adding that he had always been a most temperate man, and had never been ill before. Well, what was to be done to give him any chance of relief? I said to the assistant, 'I shall at once bleed him." This evidently excited his ridicule. 'What?' said he, 'bleed a pulseless man?' 'Yes,' said I;' 'wait and see the effect of my attempt.'

"I first procured two large pails, and got them filled with water about 100°. Having placed them at the side of the bed,

I cautiously raised him from the recumbent to the sitting position on the edge of the bed, and put each foot and leg into one of the pails. I then had two wash-hand basins nearly filled with water of the same temperature, and placed his hands and arms as deeply as I could into them. I then tied up his right arm, for the purpose of 'raising a vein.' At first, pulseless as he was at the wrist, no vein would rise, but after a minute or two a vein became sufficiently prominent to enable me to make a free incision into it; the first effect of this was that the blood flowed only drop by drop, but in a short time a small continuous stream followed, until enough blood had passed to relieve the stagnant circulation, when the stream increased, and at last it flowed *pleno vivo*—upon which my young friend's formerly sceptical countenance changed, and began to brighten with evident astonishment, and he expressed his wonderment. By this time the pulse at the wrist had become restored to considerable power, the venous livid congestion of the face had greatly lessened, and very soon it entirely passed away. I now requested the man to inspire as deeply as he could, upon which he said the pain in the chest and side was greatly lessened. I still allowed the blood to flow, until sixteen ounces had been collected in the basin, at which time he said he had no more pain, but felt extremely faint; upon which, having secured the vein, I removed him from a sitting to a recumbent position, and gave him two grains of opium; after which, having darkened the room by drawing down the blind, we left him, having directed the wife to give him nothing but warm milk, and as much as he might be disposed to take; and if he should fall asleep, by all means prevent his being awoke. All this took place about mid-day, and at six in the evening we went again to see him, when we fonnd him with a countenance bearing a natural aspect, pulse distinct and of moderate power, and about 100 per minute; his breathing very much relieved, but still more frequent than natural; but the pain in the side had returned to a slight extent, upon which I again tied up his arm, and, from the same orifice previously made in the vein, drew off, in a good stream, six ounces more blood; this entirely relieved him. I then repeated the dose of two grains of opium, and left him, having reiterated the instructions given in the morning. From this time, by implicit rest, sedative diaphoretic medicine, counter-irritation by mustard plasters on the chest, and light nutritious diet—chiefly milk—he day by day rapidly improved, so as to be able to return to his work after a fortnight's interval.

"On observing the conspicuously sudden and unmistakable result which followed the bleeding, my young friend declared, as we walked from the house, that he had learnt more of practical pathology, therapeutics and physiology, relating to the functions of the heart and lungs, from this case and treatment than he had gained by all his previous studies and observations made during the time which he had spent at the hospital, and in the course of his four years' previous apprenticeship, which he had passed in a large dispensary in a populous town in the West of England.

"Innumerable cases of the same severe type as the one described, perhaps few of the same very alarming character, have been treated in like manner, and with the same success, in my experience, and especially cases of puerperal peritonitis, of which twenty-seven have fallen under my treatment within the last fifty years, one only of the number having proved fatal. Therefore, my faith in the judicious use of the lancet has never forsaken me during the protracted period of clamor which has so long existed against it."—*Medical Times and Gazette*, Jan. 15, 1875.

[The above remarks are timely and sensible, and we predict that in ten years time the lancet will again come into general use.—Ed. Rec.]

